# Increasing Transfection Efficiency of Lipoplexes by Modulating Complexation Solution for Transient Gene Expression

**DOI:** 10.3390/ijms222212344

**Published:** 2021-11-16

**Authors:** Jaemun Kim, Ji Yul Kim, Hyeonkyeong Kim, Eunsil Kim, Soonyong Park, Kyoung-Hwa Ryu, Eun Gyo Lee

**Affiliations:** 1 Department of Bioprocess Engineering, KRIBB School of Biotechnology, Korea University of Science and Technology (UST), 217 Gajeong-ro, Yuseong-gu, Daejeon 34113, Korea; jamekn@ust.ac.kr (J.K.); jykim@kribb.re.kr (J.Y.K.); 2 Bioprocess Engineering Center, KRIBB, 30 Yeongudanji-ro Ochang-eup, Cheongwon-gu, Cheongju-si 28116, Korea; hkkim@kribb.re.kr (H.K.); eskim@kribb.re.kr (E.K.); sypark@kribb.re.kr (S.P.); khryu@kribb.re.kr (K.-H.R.)

**Keywords:** transfection, transient gene expression, complexation solution, CHO-S, DC-Chol/DOPE

## Abstract

Transient gene expression is a suitable tool for the production of biopharmaceutical candidates in the early stage of development and provides a simple and rapid alternative to the generation of stable cell line. In this study, an efficient transient gene expression methodology using DC-Chol/DOPE cationic liposomes and pDNA in Chinese hamster ovary suspension cells was established through screening of diverse lipoplex formation conditions. We modulated properties of both the liposome formation and pDNA solution, together called complexation solutions. Protein expression and cellular cytotoxicity were evaluated following transfection over the cell cultivation period to select the optimal complexation solution. Changes in hydrodynamic size, polydispersity index, and ζ potential of the liposomes and lipoplexes were analyzed depending on the various pH ranges of the complexation solutions using dynamic light scattering. The transfer of lipoplexes to the cytosol and their conformation were traced using fluorescence analysis until the early period of transfection. As a result, up to 1785 mg/L and 191 mg/L of human Fc protein and immunoglobulin G (bevacizumab), respectively, were successfully produced using acidic liposome formation and alkaline pDNA solutions. We expect that this lipoplex formation in acidic and alkaline complexation solutions could be an effective methodology for a promising gene delivery strategy.

## 1. Introduction

Transient gene expression (TGE) has become an attractive tool to produce small quantities of recombinant proteins for the development of biopharmaceutical products in mammalian host cell lines at an early stage [1]. Chinese hamster ovary (CHO), human embryonic kidney 293, baby hamster kidney, and COS cells are commonly used host cell lines for TGE [2]. Among them, CHO cells are the most widely used as a mammalian host [3], due to their high-yield protein production and established production methods [4].

For gene delivery, viral (adenovirus, retrovirus, lentivirus, etc.) and non-viral delivery systems have been developed. The non-viral delivery system, including cationic lipids, cationic proteins, and polymers are known to be an easy, safe, and relatively lower immune response methodology, although they have a lower efficiency of gene transfection [5,6,7,8]. The complexes of cationic liposomes and nucleic acids, lipoplexes, are widely used in TGE and gene therapy technologies, along with polyplexes (polymers with nucleic acids) containing cationic polymers (e.g., polyethylenimine) or biodegradable polymers (e.g., cyclodextrin, dextran, and chitosan). Using these cationic carriers, research of the treatment of genetic disease by carrying nucleotide-based agents into the body has been actively conducted in recent years [6]. Especially, the positively charged head group of cationic carriers exhibiting electrostatic attraction with phosphate group in nucleic acids is known to be related with cationic carrier-associated cytotoxicity and efficiency of gene release [9]. As efficient cationic lipids, 1,2-dioleoyl-3-trimethylammonium-propane (DOTAP), 1,2-di-O-octadecenyl-3-trimethylammonium propane (DOTMA), 3β-[N-(N′,N′-dimethylaminoethane)-carbamoyl]cholesterol hydrochloride (DC-Chol), and phosphatidylcholine lipids have been used in combination with helper lipids, such as 1,2-dioleoyl-*sn*-glycero-3-phosphoethanolamine (DOPE) or cholesterol [10,11,12]. In particular, DC-Chol and DOPE is a promising liposome combination and can be used as a highly effective nanocarrier for delivering therapeutic molecules, such as nucleic acids and drugs, into mammalian cells [13,14,15]. Furthermore, delivery systems for artificially engineered RNAs using cationic lipids have been actively studied in both academic research and clinical trials [16], and presently, an mRNA delivery system based on cationic lipids is at the center of attention because of COVID-19 vaccines [17]. Consequently, cationic lipids and polymers have potential for future use through steady research and development to overcome their various barriers.

Time to market is an important consideration in the biopharmaceutical industry. Therefore, for the past few decades, various strategies to improve transfection efficiency have been studied, including the development of new cationic carriers [18] and efficient serum-free media [19], feeding strategies, and various types of enhancers [20,21,22]. In particular, efforts to improve the transfection efficiency of cationic polymers have been made by optimizing the pH or ionic strength of the complexation solution, which is used to form a complex of cationic carriers and nucleic acids. For example, polyethylenimine (PEI), a cationic polymer, is known to modify the size, size distribution, and the colloidal stability of polyplexes, depending on the salt ion concentration [23,24] and pH of the complexation solution [25]. Furthermore, the acidification rate of polyplex-containing endosomes and the intracellular decomplexation rate of the polyplexes can be altered by adjusting the environmental pH [26]. In particular, complexation solution has a considerable effect on the physicochemical properties and intracellular pathway of polyplexes, which are the central factors that directly determine the transfection efficiency [27].

To a similar extent as in polyplexes, the properties of the complexation solution could also be important for lipoplex formation and lipofection efficiency because physical reactions, such as DNA condensation, liposome restructuring, and multiple formation, occur in the complexation solution during the lipoplex formation process [28]. Furthermore, both cationic lipids and DNA are known to be sensitive to environmental conditions [29,30,31]. Depending on the external pH, the interaction efficiency of cationic lipids changes with nucleic acids or other anionic counterparts, whereas in the case of plasmid DNA (pDNA), the structure and charge state of the DNA–counterion complex could be influenced by external ion concentrations and pH. In addition, the transfection efficiency of the lipoplex fluctuates by at least 100-fold in luciferase gene expression depending on which complexation solution used [32], and appropriate salt ions of the solution can facilitate the stabilization of lipoplexes [33]. However, these crucial factors are commonly neglected during the development of novel and efficient liposomal carriers. As a result of not considering the extent to which the complexation solution influences the transfection efficiency, saline, several serum-free media, phosphate-buffered saline (PBS), or distilled water (DW) have routinely been used for preparation of cationic liposomes and pDNA for lipoplex formation [34,35,36].

In this study, we aimed to improve the transfection efficiency of cationic liposomes in CHO-suspension (S) cells by modulating the properties of the complexation solution. We further aimed to analyze changes in physical properties of the transfection particles and observe the intracellular conformation of lipoplexes by labeling both pDNA and lipids to compare the cellular uptake and decomplexation efficiency. Finally, we evaluated our results by comparing them with the expression of green fluorescent protein (GFP), human Fc protein (hFc), and immunoglobulin G, bevacizumab.

## 2. Results and Discussion

### 2.1. Lipoplex Preparation with Cationic Lipids and pDNA

For cost-effective and high-yield protein production, cationic lipids and helper lipids have been conventionally used as an effective combination for liposome-based gene delivery. The binary lipids of DC-Chol and DOPE, which are effective in the HEK293 TGE system [14], were found to be an effective combination in CHO-S cells as well. As the ratio of constituents of lipoplexes is known to influence their physical properties, such as size, surface charge, encapsulation degree, and colloidal state and determine the transfection efficiency [37], we first obtained the optimal molar ratio of DC-Chol to DOPE and the charge ratio of nucleic acids to DC-Chol. The molar ratio of the binary lipids was determined by adjusting the molar concentration of DOPE based on that of DC-Chol, where the charge ratio was estimated by calculating the number of charges in both negatively charged pDNA and positively charged DC-Chol.

**Figure 1:**
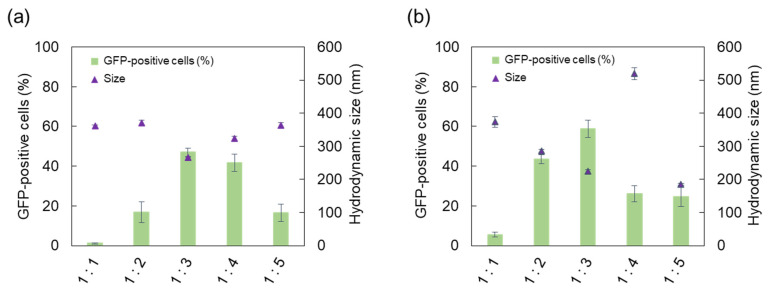
Transfection efficiency and hydrodynamic size of lipoplexes formed under different conditions of the constituent ratio: (**a**) the molar ratio of DC-Chol to DOPE; (**b**) the charge ratio of pDNA to DC-Chol. Analysis was conducted at 24 h post-transfection. Opti-MEM (Gibco) was used as the complexation solution. Among the transfected cells, GFP-positive cells were determined. The results represent the mean ± standard deviation (*n* = 3). GFP, green fluorescent protein; DC-Chol, 3β-[N-(N′,N′-dimethylaminoethane)-carbamoyl]cholesterol hydrochloride; DOPE, 1,2-dioleoyl-*sn*-glycero-3-phosphoethanolamine; pDNA, plasmid DNA.

As shown in Figure 1a, GFP-positive cells, representing the transfection efficiency, increased to maximum of 46.9% at a molar ratio of 1:3. The hydrodynamic size and PdI of lipoplexes were smaller than other ratios, indicating a relatively stable colloidal state [36]. DOPE would cover the positive charge of cationic lipids, and its excessive use would gradually lead to reduced charge repulsion between cationic liposomes, resulting in particle aggregation and a low transfection efficiency [35]. In this study, a decrease in transfection efficiency was observed when a higher-than-optimal molar ratio was used, which is consistent with a previous study [27].

In the case of the charge ratio, lipoplexes are usually formed at an excess positive ratio (more cationic moieties than nucleic acids) to condense pDNA into small cationic particles and reinforce the electrostatic interaction with negatively charged cell membranes [38,39]. In this study, various pDNA-to-DC-Chol charge ratios, from 1:1 to 1:5, were investigated with a 1:3 molar ratio of liposomes. The highest transfection efficiency was achieved when GFP-positive cells were 58.8% at a 1:3 charge ratio, with a steady decease in the percentage at greater than 1:3 ratio (Figure 1b). These high charge ratios might cause high cellular cytotoxicity through excess positive charge or irregular pDNA release from the severely internalized complexes due to a large quantity of cationic lipids [40]. Considering the sharply increased size at a 1:4 ratio, it is presumed that the colloidal stability also became unstable. Therefore, we selected 1:3 molar and charge ratios for subsequent experiments.

### 2.2. Effects of Complexation Solution on Transfection

**Figure 2:**
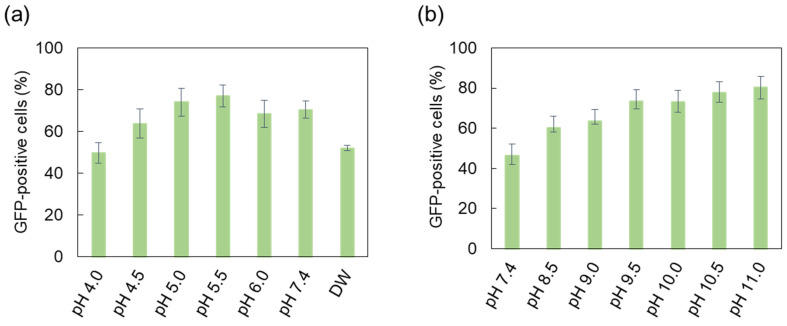
Changes in the transfection efficiency depending on pH of the complexation solution: (**a**) effects of acidic pH of the liposome formation solution; (**b**) effects of alkaline pH of the pDNA solution. GFP-positive cells were analyzed at 24 h after transfection. The results represent the mean ± standard deviation (*n* = 3). GFP, green fluorescent protein; pDNA, plasmid DNA.

DC-Chol is known to be a pH-responsive lipid due to the presence of the cationic nitrogen of its ammonium moiety, and tends to increase its net positive charge through protonation under acidic conditions [31]. Therefore, DC-Chol was prepared in an acidic or neutral buffer solution to actively facilitate binding affinity for the negatively charged payload. Furthermore, the transfection efficiency was noticeably poor when DW was used as the liposome formation solution (Figure 2a), and this could have been caused by insufficient ionic strength to screen charges and avoid particle aggregation of liposomes before interaction with pDNA [41].

As shown in Figure 2a, the weak acidic liposome formation solution (pH 5.5) presented the highest transfection efficiency, 25% higher efficiency than that with DW. However, the liposome formation solution at lower than pH 5 showed a relatively inferior transfection efficiency. The low decomplexation efficiency of lipoplexes might be a barrier at this time because excessively charged liposomes formed in a strong acidic solution could cause an extreme attraction to negatively charged pDNA, resulting in poor release of pDNA into the cytosol and a lower transfection efficiency [42]. This showed that pH of the liposome formation solution that is either too high or low can lead to a low transfection efficiency, and an intermediate pH range may be ideal for efficient transfection.

The phosphate groups of pDNA (approximate pK_a_ = 6.3) became weakly negatively charged at acidic pH, and this might negatively impact the electrostatic interaction with cationic liposomes and, eventually, the transfection efficiency [26]. Therefore, we investigated pDNA preparation solution in an alkaline pH range to facilitate appropriate charge screening for efficient electrostatic interaction when pDNA encounters cationic liposomes [43]. As shown in Figure 2b, the transfection efficiency increased with an increase in pH of the pDNA solution. The transfected cells in the pDNA solution at pH 11.0 increased by approximately 33.9% compared to that in pDNA solution at a neutral pH of 7.4 at 24 h post-transfection.

**Figure 3:**
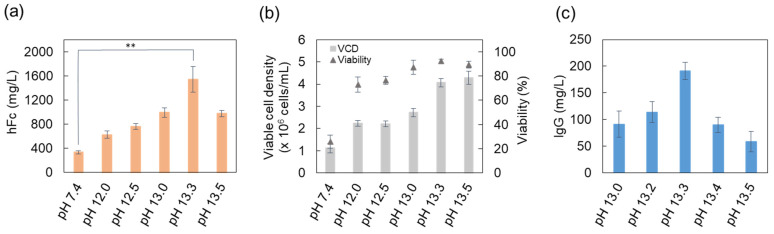
Effect of high pH values of the pDNA solution on the transfection efficiency: (**a**,**b**) the hFc protein production level and cell condition at 12 days after transfection; (**c**) the bevacizumab production level at 12 days after transfection. Using the liposome formation solution of pH 5.5, pH values higher than 12 for pDNA solution were investigated with pH 7.4 as a control. The results represent the mean ± standard deviation (*n* = 3). ** *p* < 0.01; hFc, human Fc protein; pDNA, plasmid DNA.

We further investigated the transfection efficiency at higher than pH 11.0 with the expression of hFc protein for long-term gene expression for 12 days after transfection. As shown in Figure 3a, the expression level of hFc was maximized at pH 13.3 with a concentration of approximately 1545 mg/L. Regarding cellular cytotoxicity, the cell viability was highly conserved with the increasing pH of the pDNA solution on the day of analysis (Figure 3b). In addition, 91–191 mg/L of bevacizumab was produced using the pDNA solution at pH 13.0–13.3 (Figure 3c). The ionic strength reported to shield the surface charge on the lipid structures and increase the plasmid entrapment efficiency [33], and it might have a positive effect on transfection efficiency. Ionic strength of the complexation solutions was about 50–195 mM when the weak acidic (pH 5.5) liposome formation and strong alkaline (pH 12.0–13.5) pDNA solution were mixed. In our study, the pH of complexation solutions was supposed to be a more prominent factor than the ionic strength, although the ionic strength of 100–150 mM acted as a positive factor on the transfection efficiency when pH was fixed neutrally. As a result, we observed that the pH of the complexation solution affected both the protein expression level and cell viability through TGE of three different proteins, GFP, hFc, and bevacizumab.

### 2.3. Characteristics of Liposomes, Lipoplexes, and pDNA

**Figure 4:**
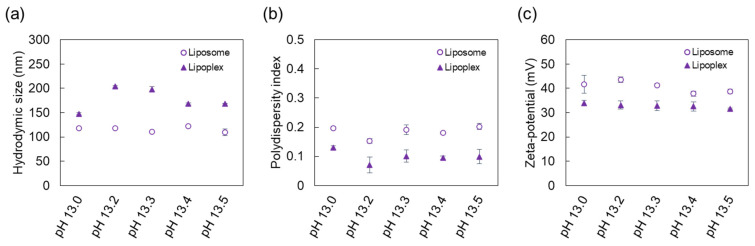
Effect of high pH values of the pDNA solution on the physical properties of liposomes and lipoplexes: (**a**) hydrodymic size; (**b**) polydispersity index; (**c**) ζ potential. The results represent the mean ± standard deviation (*n* = 3).

The physical properties of the liposome and lipoplex, known to be directly related to cellular cytotoxicity and the transfection efficiency [40], were further analyzed. As shown in Figure 4a, only liposomes without pDNA had a size of 110–130 nm, regardless of pH, whereas the size of lipoplexes were larger (up to 204 nm) in the pDNA solution at pH 13.2, indicating that the electrostatic interaction between cationic liposomes and pDNA occurred properly [44]. It is interesting to note that lipoplexes with sizes of 140–210 nm, known to be effective for in vitro transfection [45], were formed, even under alkaline conditions. Moreover, the PdI of lipoplexes was observed to be lower than that of liposomes (Figure 4b), and these low PdI values (<0.2) indicate that most complexes were formed narrowly dispersed without significant aggregation [36]. In addition, a lipoplex with a larger size and more stable colloidal state than the liposome indicates that particle interaction and stabilization were appropriate during the process of lipoplex formation [46]. Our results demonstrate that the properties of the complexation solution could influence the formation of lipoplexes and their hydrodynamic size and colloidal stability.

For accurate measurement of ζ potential, it was necessary to adjust pH of solutions containing liposomes or lipoplexes to a near neutral range. As shown in Figure 4c, the ζ potential of lipoplexes was maintained at around +30 mV, which was lower than that for liposomes, indicating that the negatively charged pDNA was internalized properly into the cationic liposomes. Therefore, positive ζ potential of the lipoplex might facilitate proper electrostatic interaction that occurs between lipoplexes and negatively charged cell membranes during transfection [47].

At such a high pH for the pDNA solution, we presumed that structural changes occurred in the supercoiled pDNA. Thus, the gel mobility shift of pDNA exposed to alkaline solution used for transfection was investigated using gel electrophoresis. When exposed to a strong alkaline solution above a pH of 13.2, a new band gradually emerged on the gel, which was distinguished from normal supercoiled pDNA. It has been reported that this alkali-denatured pDNA, which moves faster than supercoiled pDNA on an agarose gel, has a stable conformation with a rough surface and shortened contour length, using atomic force microscopy images [48]. In addition, this pDNA form is known to retain a double-stranded closed circular structure with only partially unwound parts, caused by alkali denaturation. These single strands within the circular structure are bound to each other, and a reversible form that can be restored into double strands when exposed to a low pH environment [49]. pDNA bound to cationic liposomes might be exposed to an acidic environment of pH 5.0–6.0 in endosomes and lysosomes following endocytosis [50], which could be the ideal environment for renaturation of alkali-denatured supercoiled pDNA into its native form.

A higher compaction degree of the pDNA could positively influence the interaction between cationic lipids and pDNA, and thus the physical properties of the lipoplex [51]. Similarly, we hypothesized that the compact conformation of alkali-denatured pDNA may contribute to effective internalization into cationic liposomes, resulting in stable lipoplex formation as previously observed with dynamic light scattering.

### 2.4. Cellular Uptake and Decomplexation of the Lipoplex

**Figure 5:**
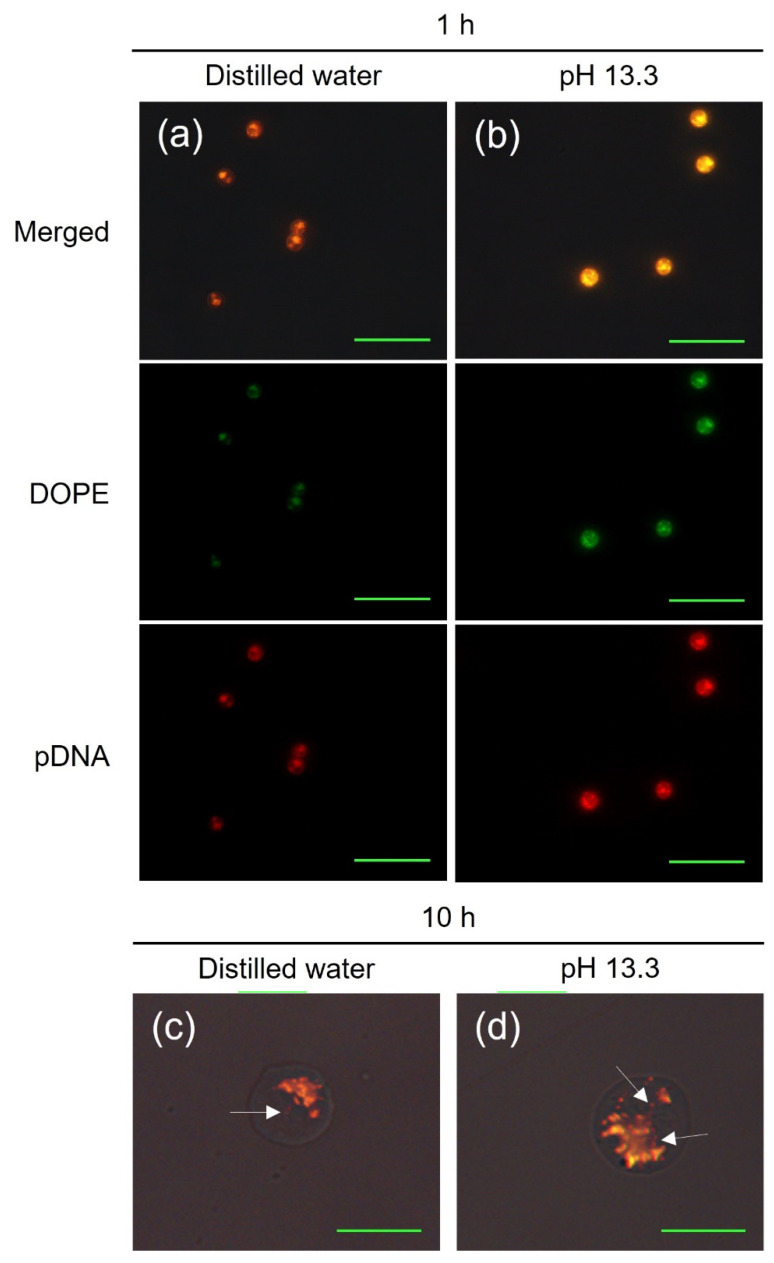
Fluorescence analysis of transfected cells to compare the intracellular distribution of pDNA and DOPE between DW and the pDNA solution of pH 13.3. Fluorescence images (**a**,**b**) showing the quantity and colocalization (orange) of Cy5-labeled pDNA (red) and Atto 532 dye-labeled DOPE (green) at 1 h post-transfection. Scale bar = 50 μm. At 10 h post-transfection, the fluorescence images (**c**,**d**) of transfected cells showed intracellular naked pDNA (red) separated from DOPE (indicated by white arrows). Scale bar = 10 μm. DOPE, 1,2-dioleoyl-*sn*-glycero-3-phosphoethanolamine; DW, distilled water; pDNA, plasmid DNA.

To account for the increased transfection efficiency in alkaline pDNA solution in terms of cellular uptake and decomplexation of lipoplexes, DW was set as a control, and fluorescence analysis was performed using Cy5-labeled pDNA and Atto 532 dye (related to Rhodamine 6G)-labeled DOPE. At 1 h post-transfection, lipoplexes successfully entered the cells in both DW and pDNA solution of pH 13.3, while pDNA was fully formed with DOPE lipids (Figure 5a,b). Analysis of pDNA fluorescence area using ImageJ software revealed that more pDNA entered cells at a solution of pH 13.3 than that in DW (data not shown), indicating that the use of alkaline pDNA solution allowed more pDNA bound to cationic liposomes to enter the cells. Even with the naked eye, the difference in fluorescence intensity was clear. This may have been due to the compact size of alkali-denatured pDNA, leading to efficient formation of the lipoplex, more interaction, and a lower PdI. 

The lipoplexes are known to be transferred to the cytosol through the cell membrane via micropinocytosis and clathrin-mediated endocytosis [52], and escape from endosomes through fusion with the endosomal membrane to avoid lysosomal degradation [53]. Subsequently, pDNA should enter the nucleus and dissociate from cationic liposomes for proper expression [54]. We investigated and compared this decomplexation efficiency of lipoplexes at 10 h post-transfection, which is the time point when endocytic processes, including endosome escape and lysosomal and cytosolic degradation of lipoplexes, are completed [53,55]. As a result, a large quantity of naked pDNA that was dissociated from DOPE was observed in cells (Figure 5c,d). Analysis of three individual images, containing a total of 122 and 147 cells (DW and pDNA solution of pH 13.3, respectively), confirmed that the red (pDNA)/green (DOPE) fluorescent area ratio was 23.1% higher in the pDNA solution of pH 13.3 than that in DW, indicating that there was a difference in decomplexation efficiency of lipoplexes.

**Figure 6:**
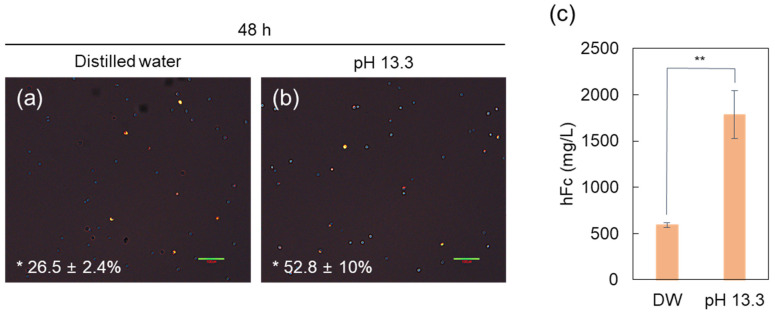
Fluorescence images (**a**,**b**) showing pDNA-positive cells at 48 h post-transfection. The number of cells containing pDNA among the cells was calculated using ImageJ software, and the percentage of pDNA-positive cells indicated in images is the average values of three individual images. Scale bar = 100 μm. The hFc protein production (**c**) at 12 days post-transfection. The results represent the mean ± standard deviation (*n* = 3). ** *p* < 0.01; hFc, human Fc protein; pDNA, plasmid DNA; DW, distilled water.

The intracellular decomplexation of lipoplexes, a prerequisite for the eventual transcription of genes in the nucleus, is considered a limiting factor to lipofection [40,42]. Therefore, improvement in decomplexation efficiency of lipoplexes is crucial for increasing the transfection efficiency [56]. In the case of PEI, it has been reported that a weak attraction between PEI and pDNA within the polyplex can facilitate efficient pDNA release from cationic polymers into the cytosol, thereby increasing transfection efficiency [26]. In this study, the alkaline pDNA solution was supposed to cause partially deprotonated portions inside the lipoplex, which are known to weaken the cationic lipid/pDNA attraction, thus aiding the ready release of pDNA from cationic liposomes [54]. As shown in Figure 6a,b, the quantity of pDNA-positive cells at 48 h post-transfection was evaluated by analyzing three separate images. The pDNA-positive cells or transfection efficiency (%) were 2-fold higher in solution of pH 13.3 than that in DW. Furthermore, the percentage of cells was proportional to pH values of the pDNA solution. This outcome led us to hypothesize that the fate of the pDNA, which is influenced by decomplexation efficiency of cationic liposome/pDNA, can be effectively controlled by modulating pH of the complexation solution. Consequently, the pDNA solution of pH 13.3 showed a 3-fold higher level of hFc protein expression (1785 mg/L) than DW (Figure 6c).

As a result, the properties of the complexation solution could affect not only the lipoplex formation process, but also the intracellular fate of lipoplexes, which is related to the transfection efficiency. In addition, a larger quantity of intracellularly absorbed lipoplexes and effective intracellular decomplexation could both be important to increase the transfection efficiency. However, whether alkali-denatured supercoiled DNA was efficiently expressed in cells is unclear although it was reported that renatured pDNA can be expressed appropriately [49]. To reach a clearer conclusion, a structural analysis of the lipoplexes should be performed in the future as the nanostructure of lipoplexes, including large unilamellar and multilamellar complexes, can change depending on the conditions of the lipoplex formation process and is related to the transfection efficiency [8,28]. In addition, a more accurate analysis to compare the decomplexation efficiency of complexes is required. Therefore, these factors need to be considered in future studies for a better understanding of the effects of the complexation solution on the efficiency of lipofection. Nevertheless, our results contribute to the proof of concept that modulation of the properties of the complexation solution can break the barriers in lipofection and improve the transfection efficiency.

## 3. Materials and Methods

### 3.1. Cell Culture

ExpiCHO-S cells (CHO-S, Gibco, Waltham, MA, USA) were routinely cultured in ExpiCHO Expression Medium (Gibco) at a seeding density of 0.3 × 10^6^ cells/mL in 125-mL disposable polycarbonate Erlenmeyer flasks (Corning, Corning, NY, USA) in a humidified 8% CO_2_ incubator at 110 rpm. The cells were subcultured every 3 to 4 days. The cell density and viability were determined with a 300-µL sample using a Cedex HiRes Analyzer (Roche, Basel, Switzerland).

### 3.2. Transfection Reagents and Expression Vectors

DC-Chol and DOPE (both from Sigma; St. Louis, MO, USA) were dissolved in pure ethanol at concentrations of 5 mg/mL and 4 mg/mL, respectively, and used as the lipid combination for lipofection. Three vectors expressing hFc (5211 bp), bevacizumab (8993 bp), and GFP (3774 bp) were used. The hFc and bevacizumab sequences were cloned into the *Nhe*I and *Xho*I sites of the pcDNA3.1(+)/Zeo vector (Invitrogen, Carlsbad, CA, USA). Wood-chuck hepatitis virus PRE sequences (WPRE) were placed upstream of the polyadenylation signal sequence to create the WPRE vectors. The pDNA was amplified in *Escherichia coli* DH5α cells, purified using EndoFree Plasmid Maxi and Giga Kits (Qiagen, Germantown, MD, USA), and finally dissolved in Tris-EDTA buffer (10 mM Tris-HCl, 1 mM EDTA; pH 8.0).

### 3.3. Transfection

The acidic liposome formation solution at the desired pH ranges (pH 3.5 to 5.5) was prepared by dissolving an appropriate concentration of sodium acetate (4.8–89.7 mM) and acetic acid (10.3–95.2 mM) in distilled water. The pH values of alkaline pDNA solution (up to pH 13.5) were adjusted with sodium carbonate buffer (9–87.5 mM) or sodium hydroxide solution (10–300 mM) and measured using Orion 2-Star Benchtop pH Meters (Thermo Fisher Scientific, Waltham, MA, USA). Transient transfection was conducted according to the manufacturer’s protocol. The CHO-S cells were prepared at a density 6.0 × 10^6^ to 7.0 × 10^6^ cells/mL and centrifuged at 300× *g* for 5 min. Subsequently, the cells were resuspended at a density of 3.5 × 10^6^ to 4.0 × 10^6^ cells/mL in fresh pre-warmed medium. The next day, before transfection, the viable cell density was 9.0 × 10^6^ to 10.0 × 10^6^ cells/mL and adjusted to the appropriate density by addition of fresh pre-warmed medium. Transfection was performed at a culture scale of 20 mL. Lipoplexes were prepared by separately diluting DC-Chol/DOPE and pDNA (20 μg) in 1 mL of ice-cold complexation solution prepared in different tubes and then adding the lipids to the pDNA. Liposome and lipoplex formation steps had incubation times of 2 and 4 min, respectively. The lipoplexes were then added to a cell culture flask. ExpiFectamine CHO Enhancer and Feed (both from Gibco) were added to the transfected cells at 20 h post-transfection when hFc and bevacizumab were produced.

### 3.4. Analysis of Protein Titer and GFP-Positive Cells

Samples of more than 300 µL were collected and centrifuged at 3000× *g* for 5 min to analyze concentrations of produced proteins hFc and bevacizumab. The quantity of recombinant protein in the 250-µL supernatant was determined using Cedex Bio HT Analyzer (Roche, Mannheim, Germany). The cells transfected with the GFP-expressing vector were cultured for 24 h and harvested. In the samples diluted to 0.3 × 10^6^ cells/mL using PBS, GFP-positive cells were analyzed and calculated in 5000 cells using Guava easyCyte HT System (Millipore, Burlington, MA, USA).

### 3.5. Dynamic Light Scattering

Samples were freshly prepared just before measurement, diluted 20× in PBS, and then adjusted to near neutral pH. The hydrodynamic size, polydispersity index (PdI), and ζ potential of liposomes and lipoplexes were analyzed using Zetasizer Nano ZS (Malvern Panalytical, Malvern, UK), operated at a 90° scattering angle and with a 633-nm laser source at room temperature.

### 3.6. Gel Electrophoresis

Agarose gel (0.8%) was prepared in Tris-acetate-EDTA buffer to confirm the gel mobility shift of pDNA, and electrophoresis was conducted at 100 V for 25 min with 2 μg of pDNA in 50 μL solution. The pDNA was visualized using RedSafe Nucleic Acid Staining Solution (iNtRON Biotechnology, Seongnam-Si, Korea).

### 3.7. Fluorescence Imaging

Small-scale transfection using 24 square deep well plates (Applikon Biotechnology, Schiedam, The Netherlands), Atto 532 DOPE (Sigma-Aldrich, Saint Louis, MO, USA), which was fluorescently labeled with the Atto 532 dye, and fluorescent labeled hFc pDNA with a Label IT Tracker Intracellular Nucleic Acid Localization Kit, Cy5 (Mirus Bio, Madison, WI, USA) was performed to analyze the intracellular conformation of the complexes. The transfected cells were maintained in a humidified 8% CO_2_ incubator at 37 °C and 240 rpm. The transfected cells were diluted to a concentration of 1 × 10^6^ cells/mL for analysis of fluorescence, followed by induction of excitation at fluorescence wavelengths of 530 nm and 625 nm under a F1-CIS fluorescence microscope (Nanoscope Systems, Daejeon, Korea). ImageJ software (National Institutes of Health, Bethesda, MD, USA) was used for analysis of fluorescence in each image.

## 4. Conclusions

In this study, the ratio of the lipoplex constituents, acidic and alkaline complexation solution, and lipoplex formation and decomplexation were investigated to improve the transfection efficiency of pDNA TGE using cationic liposomes. Appropriate ratios of DOPE and cationic lipids in lipoplexes were found to be a critical factor for lipoplex formation and transfection efficiency, which could be further improved when acidic liposome solution and alkaline pDNA solution are used. An appropriate ionic strength and acidic-alkaline pH selection of the complexation solution might best contribute to the efficiency of the electrostatic interaction between cationic liposomes and pDNA, and the properties of the lipoplex. Consequently, up to 1785 mg/L and 191 mg/L of the hFc protein and bevacizumab were produced, respectively, and there was a 5-fold higher production of the hFc protein compared to that in control (PBS). We suggest that this efficient transfection resulted from changes in the physical properties of the lipoplex. Additionally, partially deprotonated portions inside the lipoplex that can be activated in an alkaline solution might improve the decomplexation efficiency of lipoplexes and the intracellular survival rate of pDNA, which may be caused by protection against endosomal and cytosolic degradation. Therefore, this approach is a promising strategy, applicable to other gene delivery systems using cationic lipids.

## Data Availability

The data presented in this study are available on request from the corresponding author.
